# Qualitative Study of Health Information -Seeking Barriers among Mastectomy Patients

**DOI:** 10.31557/APJCP.2020.21.11.3185

**Published:** 2020-11

**Authors:** Masoomeh Latifi, Meghdad Sedaghat, Nilofar Barahmand, Fatemeh Fahimnia, Leili Allahbakhshian Farsani

**Affiliations:** 1 *Mother and Child Welfare Research Center, Hormozgan University of Medical Sciences, Bandar Abbas, Iran. *; 2 *Department of Internal Medicine, Imam Hossein Hospital, Shahid Beheshti University of medical science, Tehran, Iran. *; 3 *Scientometrics Office, Vice Chancellery of Research, Shiraz University of Medical Sciences, Shiraz, Iran. *; 4 *Department of Information Sciences and Knowledge Studies, Faculty of Management, Tehran University, Tehran, Iran. *; 5 *Vice-Chancellery for Research and Technology, Isfahan University of Medical Sciences, Isfahan, Iran. *

**Keywords:** Health information, seeking barriers, women, breast neoplasm, mastectomy

## Abstract

**Background:**

Health information-seeking behavior (HISB) plays a key role in self-care management, promoting quality of life and improving health. However, some individual and contextual barriers hinder women undergoing mastectomy access to needed information. Identifying and removing health information-seeking barriers for these women undergoing mastectomy can lead to improving their health outcomes. Therefore, the aim of this study was to identify the health information-seeking barriers for women with breast cancer after mastectomy.

**Materials and Methods:**

This was a conventional qualitative content analysis in which the participants were selected through purposive sampling based on the study inclusion criteria from two hospitals of Shahid Mohammadi and Persian Gulf and Chemotherapy Center of Omid in Bandar Abbas. The study population consisted of 17 women with breast cancer after mastectomy. Data were collected through semi-structured face-to-face interviews.

**Results:**

Seven main themes were introduced as three individual barriers, including fear, shame and embarrassment and inadequate health literacy and four contextual barriers of economic status, physicians and medical staff, lack of accessibility of information sources and the behavior of those around them that were the underlying factors to explain the barriers of health information seeking in mastectomized women.

**Conclusion:**

The results of this study emphasize the need for further attention from Iranian authorities to health care, especially women’ health care institutions, to reform the health system and remove their health information -seeking barriers.

## Introduction

Breast cancer is one of the most common cancers among Iranian women, and also the statistics of Iran cancer registry center shows an increasing trend in breast cancer incidence over the recent years (Fazel et al., 2019). The mastectomy is a way to treat breast cancer by removing the breast with or without the lymph nodes. Women who undergo mastectomy experience surgery, physical defects and physical and mental threats of cancer which can lead to psychological trauma, including depression and anxiety, changes in lifestyle, fears and concerns about body image, recurrence, and even death (Najafi et al., 2005). However, some studies have shown that access to appropriate, high-quality information can lead to their self-care improvement and not only decrease the impacts of these threats but also lead to their coping with disease, better interaction during and after treatment, reducing anxiety and mood disorders success in self-care, improving communication with family and having a good life after surgery (Gumus et al.,2010; McLachlan , 2009; Miyashita et al., 2015; Recio-Saucedo et al., 2016). Health information-seeking behavior (HISB) can help women undergoing mastectomy to access the information they need. The HISB is defined as the sum of activities attempted by an individual after determining their health information needs to meet them, indicating how they find and use information about their illness (Jung, 2014). Research has shown that people face numerous barriers that make it difficult for them to access their health information needs. Lambert and Loiselle (2007) reviewed articles related to HISB and identified two main types of barriers: (i) individual barriers and (ii) contextual barriers. Individual barriers include demographic characteristics, psychological factors, health beliefs, language barriers, perceived self-efficacy, and contextual barriers comprise media’s inattention to providing information, inadequate health system support, costs and social and cultural factors (Lambert and Loiselle, 2007).

Several studies have been conducted on the health information-seeking barriers for women with cancer after mastectomy. Nakashima et al., (2012) identified the most important individual barriers to seeking health information in Japanese women with breast cancer, including the need for the person’s great effort to access to information, worrying about information quality, lack of time, and lack of understanding medical information (Nakashima et al., 2012). Liao et al., (2014) identified other barriers, such as fear of receiving information about illness, depression, low self-esteem, post-surgical trauma due to amputation of breast, motherhood, and susceptibility to mental and emotional disorders. Age is also among important barriers to women’s health information seeking. Older-aged individuals are less likely to seek health information (Liao et al., 2015). Matthews et al., conducted a study on African-American breast cancer patients and found that fear of social stigma and religious beliefs were the most significant barrier to seeking health information in ethnic group (Matthews et al., 2002).

Regarding the contextual barriers, the results obtained from a study conducted on 329 Greek women with breast cancer showed that the majority of patients are not active in seeking health information during course of disease because relationships between physician and patient were largely paternalistic in Greece, in which patients often assigned different decisions to the physician and the medical staff (Almyroudi et al., 2011). Other studies have pointed to the lack of accessibility of information sources, the lack of necessary information about services, and the lack of awareness of health information providers about patients’ preferences for seeking their health information needs (Liao et al., 2015; Vaziri et al., 2014). A broad look at the research suggests that very few studies have been conducted on health information-seeking barriers for female breast cancer patients in Iran. Therefore, due to the lack of research and increased trend of breast cancer incidence in Iran, a study on health information-seeking barriers for female breast cancer patients in Iran seems necessary. Considering recent policies of cultural and social council for women and family on women’s equal right to the enjoyment of the highest attainable standard of health, necessity of this study becomes more evident. Increasing central role of women in self-care, increasing access to information (in particular via the national and mass media), and high-quality health-care services to meet their needs in different periods of life and removing economic, social and cultural barriers affecting women’s health are among major strategies of these policies (latifi et al., 2019). Implementing these strategies without achievement of research findings on health information-seeking barriers for women with breast cancer after mastectomy would not be associated with expected outcomes. Therefore, the present study was conducted to identify the health information-seeking barriers for women with breast cancer after mastectomy in Hormozgan Province, Iran. According to reports by Ministry of Health and Medical Education (Iran), and Shojaei et al., (2020) research results Hormozgan Province is one of the most deprived regions regarding access to medical and health services and interventions, and it is in the last rank in terms of developing health and medical issues in Iran. Conducting studies in this region can provide necessary information for authorities for health information services and interventions on breast cancer to remove the health information-seeking barriers and may provide an opportunity for conducting future research works in other regions. 

## Materials and Methods

This was an applied qualitative research and the reason of choosing method was due to both a research gap and a lack of empirical studies on the health information-seeking barriers for post mastectomy women, especially in Iran. Inductive and exploratory approaches to qualitative method help identify the hidden dimensions of the phenomenon under study. On the other hand, because of the basic differences between cultural and social contexts, a qualitative research method was chosen in the present study rather than applying theories and quantitative research method. This research was carried out the conventional content analysis to extract reliable results data and identify the themes of obvious and hidden text from the internalized data (Hamilton and Finley, 2019). Participants were selected through purposive sampling based on the study inclusion criteria. The study inclusion criteria were women with breast cancer who had undergone mastectomy by the surgical removal of one or both breasts, partially or entirely. During the study, there was no accurate statistics on the women underwent mastectomy in Hormozgan province, and a list of them from Shahid Mohammadi and Persian Gulf hospitals and Omid Chemotherapy Center in Bandar Abbas was provided after repeated efforts. Of the 25 women identified, six were left prior to initiation of the study the study and two were withdrawn during the study. The study population consisted of 17 women with breast cancer after mastectomy. Data were collected through semi-structured face-to-face interviews in Winter 2015.

An interview guide for this study was prepared based on the research objectives, researchers’ experiences in the field of information behavior, the study of relevant research, and pretest interview with one of the participants whose key questions were as follows: “What barriers did you face in accessing your information needs?” The answers were then guided interview process.” For example, the researchers asked one participant who mentioned the fear of receiving information: you say, “you are afraid of asking question about the disease, can you please explain more about it”. The participant was asked to clarify with an example if necessary. For example, you say, “any questions don’t have to ask doctor, can you give an example of it”. Interviews lasted 45–75 minutes, and face-to-face interviews were conducted in hospitals, physicians’ offices and at home of the participants as appointments were previously set on the phone, and the participants allowed the recording of the interviews and then they were typed in MS word processor software. Data collection continued until saturation was achieved. The interviews were transcribed verbatim and coded for analysis. The key concepts and codes were put into sentences and paragraphs and classes created and finally, the themes were extracted (Elo and Kyngas, 2008).

For example, statements that reflected fear of recurrence of the disease or there was a failure to respond during the course of treatment were classified as fear of the ineffectiveness of the course of treatment, and also statements that reflected fear of the consequences of disease and side effects of chemotherapy were classified as fear of the post-operative restrictions, and finally these subcategories were put into the larger category of fear. To evaluate validity and reliability of the data, Guba and Lincoln’s criteria, namely the credibility, dependability, confirmability, transferability were considered (Guba and Lincoln, 1994). To evaluate reliability, investigator triangulation and constant comparison methods were used, so that the expertise have appropriate time for data collection, the use of breast cancer surgeons’ viewpoints and views in analysis and agreement with participants about themes obtained were procedures used for ensuring validity and reliability of the data. To assess confirmability, data were given to two breast cancer specialists and their viewpoints were compared and analyzing of their viewpoints that showing many agreements were approved. Transferability was also achieved by the data explained in depth. Before data collection, ethical principles, such as informed written consent, anonymity, confidentiality, and privacy and allowed to leave the study at any time were incorporated into this study.

## Results

*Demographic characteristics of participants*

A total of 17 women aged 37-65 years participated in the study. Their post-operative times varied from 8 to 60 months; 16 participants were married and all had children and 14 ones were housewives. Four cases had low economic status, ten ones had moderate economic status, and three individuals had a high economic status. Ten participants had high school education, four had high school diploma and three had bachelor’s degree.

*Health information seeking barriers*

Results showed that seven main themes emerged: three individual barriers, such as fear, shame and embarrassment and inadequate health literacy and four contextual barriers, such as economic status, physicians and medical staff, lack of accessibility of information sources and the behavior of those around patients. 

*Fear*

Participants had fear of receiving information about their illness. In this regard, three sub-themes emerged: (i) fear of receiving bad information about the disease, (ii) fear of the ineffectiveness of the course of treatment and (iii) fear of the post-surgical restrictions. Regarding the fear of receiving bad information about the disease, participant 1 says, “Because of cancer, doctor removed my uterus along with one of my ovaries long time ago, it was a bad experience. When I found that my breast need to remove, this news startled me, now, I am afraid of death due to breast cancer, and that is why I personally would have preferred that I know less”. On the other hand, fear of the ineffectiveness of the course of treatment caused participants’ reluctance to seek information. Participant 12 says, “At 32 my sister was diagnosed with breast cancer and died at the age of 35. I accompanied her in the whole course of treatment. Now, when I visit the doctor’s office, I’m full of fear. I’m afraid, the doctor will say, it’s too late. Delayed treatment has allowed your illness to spread to another part of the body”. Fear of the post-surgical restrictions was another reason for the participants’ reluctance to seek information. Participant 17 says, “Doctor removes my left breast and I need chemotherapy. Although I am trying to ask my doctor to tell me that to which extent would chemotherapy be damaging to my body? I’m afraid to ask. Even when I visit the doctor’s office and I see other women talk about their illness, their operation or their problems, I would prefer not to hear anything about them due to fear”.

*Shame and embarrassment*

Participants expressed that their sexual information needs were one of the most important needs that did not meet, and they did not want to talk about them because of shame and embarrassment, and physicians and other health care providers did not provide information in this regard. Participant 12 says, “I experience a decrease in sexual desire after the operation. I don’t know what causes it. This happens because of breast removal or it is natural, and whether all the women experience sexual problems after surgery or not? I try to tell my doctor several times, but I feel embarrassed and ashamed”. Participant 8 says,” It’s hard enough to tell my doctor about sexual problems. A majority of women experience sexual problems after surgery. Doctors know such problem better than anyone. Doctors can ask us whether you are having problems with sex or not? It’s easier to talk with my doctor about my sexual problems “.

*Inadequate health literacy*

The participants expressed that inadequate health literacy was one of health information -seeking barriers. They believed that there was a need to train postoperative care before they were discharged from hospital because individuals with inadequate health literacy were finding it difficult to seek, understand, and use information. Participant 5 says, “We have a family history of high blood pressure. I have some problems when I’ve taken Erythromycin after the operation. I just install the pharmacy software on my mobile phone. Sometimes I read books, but I can’t understand these high-level materials. Participant 3 also says, “I’ve read some books, I know that many physical therapists recommend daily stretching exercises after mastectomy. My doctor recommends the same way, but their images or even their large descriptions can’t help the individuals’ understanding. I think not everyone possesses this knowledge “. 

*Economic status*

Although the participants preferred to seek information from their physician, there was a delay in in referral of them to the physician because of their financial problems. In this regard, participant 6 says, “250,000 rails for fortnightly visit to doctor for my husband, who works in the firm as a worker and has no insurance coverage, is a lot of money. My husband can’t afford to pay for my chemotherapy “.

*Physicians and medical staff*

Physicians and medical staff-related barriers came with two sub-themes such as not receiving enough information and the use of medical terms. Although the participants expressed that they had a trust in information that could be provided from physician, they were dissatisfied with not receiving enough information which was one of health information -seeking barriers. Participant 13 says, “Despite the high costs of their visit, doctors don’t always spend enough time with their patient, I ask my doctor for the simultaneous or delayed reconstruction. My doctor doesn’t really want to completely answer my questions. We need to know the answer to our questions. Whenever I visit his office, I’m not in his room only five minutes. When I ask every question, my doctor just shakes her head and tells me it is normal, without giving any further explanation on it”. On the other hand, the participants expressed that another barrier to seeking health information was the overuse of medical terms by physicians. Participant 9 says, “My doctor tell me that you have to receive adjuvant therapy to prevent metastasis formation, when I ask my doctor, I don’t understand what you are saying. Is it dangerous? Am I going to die? These words are difficult to understand. Well, doctor tells me that I have advanced cancer “.

*Lack of accessibility of information sources*

The participants expressed that another barrier to seeking health information was lack of accessibility of information sources with two sub-themes, such as the lack of printed sources and the lack of television programs. Participants were dissatisfied with a shortage of printed information sources in hospitals or even physicians’ offices. Participant 16 says, “We can’t remember many medical advices, what should we eat? What should we avoid to eat? What should we do? What should we avoid to do? We can’t. We don’t have access to the patient’s information booklet. It would be better if we have access to it. If we forget the doctor’s advices, this booklet can help us. We do not have to visit the doctor’s office each time “. On the other hand, some participants were dissatisfied with the lack of television programs on breast cancer which was another barrier to seeking health information. Participant 7 says, “There is a television channel about health in which every program focuses on one single disease and a doctor is invited to talk about it, but so far, this TV channel has not shown any program on types of mastectomies used in the surgical treatment of breast cancer to answer our questions about breast cancer”.

*The behavior of those around patients*

Some participants also expressed that those around them did not know how to behave with cancer patients and sometimes the behavior of those around patients was so annoying that patients would prefer not to receive their help and support and avoid asking their health information needs. Participant 10 says, “At a party, I ask my sister-in-law’s girl, who is a doctor, for the use of tamoxifen and its side effects. At this moment my relative says, she has breast cancer. Perhaps she doesn’t have any bad intentions, but I am immediately irritated and annoyed and would prefer not to ask the rest of my questions “.

## Discussion

Although one of factors affecting self-care was health information seeking, our results showed that three individual barriers (such as fear, shame and embarrassment and inadequate health literacy) and four contextual barriers (such as economic status, physicians and medical staff, lack of accessibility of information sources and the behavior of those around patients) were identified as health information-seeking barriers for women with breast cancer after mastectomy. According to the participants’ experiences, fear of receiving information about their illness was one of the most important barriers to heath information seeking, so that they avoided knowing more about their illness and conditions. In their study, Liao et al., (2014) also demonstrated that fear as a psychological factor is the most important factor that affects information-seeking behavior. Perhaps, because the word cancer and its negative notion makes most of us tremble in fear and most people with cancer think that cancer causes their premature death. Moreover, the results of the study of Liao et al. showed that women have in fact suffered post-operative trauma after the mastectomy which is an operation to remove one or both breasts and their fear and anxiety causes them to avoid seeking health information (Liao et al., 2014). Similar results were obtained in the present study. According to the participants’ experiences, another individual barrier to health information seeking was shame and embarrassment. This theme seemed more related to getting about information about the sexuality, so that the participants were reluctant to ask and meet it despite their information needs. However, theme of shame and embarrassment was not investigated in the studies reviewed (Jung, 2014; Nakashima et al., 2012 ;Liao et al., 2014), except for the study of Matthews et al., (2002) who investigated the African American ethnic group. It is likely that the discrepancy between results may be attributed to the unspoken nature of sexuality in Iranian culture due to shame and embarrassment in which women feel ashamed of their sexuality and sexual needs.

Another individual barrier to health information seeking was inadequate health literacy that could hinder the use of sources, the use of the Internet and electronic sources, and the understanding of medical terms and terms associated with the disease. Since the majority of the participants in this study had high school education and a high school diploma, this result was not surprising. On the other hand, the majority of participants did not have a high economic status, which was one of their health information -seeking barriers. This finding was not consistent with findings from Liao et al., (2014) and Nakashima et al., (2012). It should be noted that in studies conducted on health information-seeking behavior, economic status is one of the factors that influence HISB, so that individuals with lower economic status are less likely to seek information (Brashers et al., 2002; Czaja et al., 2003). This may justify the aforementioned difference, as the present study was conducted in one of the most deprived areas in Iran, the majority of participants had a low or moderate economic status and did not refer to a physician because of costs. However, physicians and medical staff inhibit them to seek health information when they refer to a physician. Physicians and medical staff did not spend enough time with their patient, and on the other hand, due to the use of medical terms by physicians, the participants did not benefit from the information provided and many of their questions remained unanswered. A lack of attention to the patients’ preferences and information needs was another factor that had been identified the theme such as physicians and medical staff as one of health information seeking barriers. Liao et al., (2014) also reported the same barrier. Despite the abovementioned barriers, the participants did not have access to appropriate information sources related to their literacy levels, information needs and preferences. Similar results were obtained in study of Liao et al., (2014).

The behavior of those around them was identified as one of health information -seeking barriers for women with breast cancer after mastectomy. However, studies conducted on cancer information seeking behavior have shown that supportive others, the individual’s social relation network, and information support from family and friends are main motivational factors for patients to seek heath information (Czaja et al., 2003; Wang et al ., 2013).

However, our study has some limitations. One of the limitations is the lack of a systematic database relevant to women with breast cancer after mastectomy in Hormozgan province, which may affect the study sample. The researchers frequently referred to hospitals and the Bandar Abbas Chemotherapy center and tried to solve the limitations mentioned above. On the other hand, the present study was conducted in the Hormozgan province which is one of the most deprived areas in Iran and its findings cannot be generalized to other areas. Conducting similar studies in other areas will allow comparisons with the results of our study 

In conclusion, this study aimed to identify the health information-seeking barriers for women with breast cancer after mastectomy. The results showed that despite the importance of access to health information in order to cope with challenges caused by breast cancer following mastectomy, women would face individuals and contextual barriers to seek health information. our results revealed that three individual barriers (such as fear, shame and embarrassment and inadequate health literacy) and four contextual barriers (such as economic status, physicians and medical staff, lack of accessibility of information sources and the behavior of those around patients) were identified as health information -seeking barriers for women who had undergone mastectomy for breast cancer. Although we cannot generalize the results, they appear to be the point of departure for implementing policies and strategies for Cultural and Social Council for Women and Family in order to increase women’s access to information (in particular through the national mass media), and high-quality health care services tailored to their needs in different periods of life and remove economic, social and cultural barriers affecting women’s health.

*Suggestions for Future Research*

According to the results of this study, we will have to take the necessary steps remove all health information barriers for women with breast cancer after mastectomy. As the first step, providing counseling and psychological services for women can help them reduce their fears, anxieties, worries, and manage their feelings or emotions after the surgery helps them in order to seek information about their illness and self-care. In their study, Carr et al., (2014) showed that providing visual information about women’s body image after the mastectomy can reduce trauma; Therefore, it is advisable to make women aware of this issue by using Carr et al.’s strategy and providing visual information, which in turn can have significant effects on reducing their fears and worries in order to encourage them to seek information. On the other hand, because lack of awareness of sexual problems after surgery can result in sexual dissatisfaction between couples and the deterioration of family relationships and the quality of their family relationships (Carr et al., 2019), it is therefore recommended that the treatment groups should not focus only on the patients’ survival and maintaining their lives. Accordingly, it is essential that health care providers take into account sexual issues and provide the necessary training to them and their partners with respect to the prevailing values and attitudes in the community. Moreover, women and local professionals can provide the counseling and psychological services which can be helpful in this regard. Despite health information -seeking barriers, such as inadequate health literacy, the economic status, lack of accessibility of information sources and physicians and medical staff, it is imperative that Health System in Iran provide strategies for the well-being of low-income women undergoing mastectomy for breast cancer and offer free consultation and health information services, especially through medical centers, cancer patient support associations and relevant organizations. Providing health information outreach program can also be effective. It is also useful to provide information services through written and illustrated sources to patients and their families. However, given the inadequate health literacy as one of health information -seeking barriers for patients, it is necessary not only to provide information sources tailored to their preferences, needs and literacy levels, but also to facilitate and opportunities for them or their families to promote their literacy levels and use available information sources effectively. On the other hand, there is a need for fundamental changes in the structure of the health system in Iran so that the culture of providing services with an emphasis on interpersonal communication skills, attention to patients’ needs, preferences and literacy levels and encouraging them to participate in medical interventions and self-care can spread among physicians and medical staff. Radio and TV, as a relatively cheap and easily accessible source of transmitting information, can play an important role in the removal of health information -seeking barriers above mentioned. Also, formation of support groups in relevant organizations, accessible local centers such as mosques and libraries can be effective in exchanging information and providing emotional and spiritual support for both the patient and their families
